# Term and Preterm Birth Initiation Is Associated with the Macrophages Shifting to M1 Polarization in Gestational Tissues in Mice

**DOI:** 10.3390/biology11121759

**Published:** 2022-12-04

**Authors:** Yali Shan, Shiping Shen, Jing Long, Zhengshan Tang, Cichun Wu, Xin Ni

**Affiliations:** 1 Department of Gynecology and Obstetrics, Xiangya Hospital Central South University, Changsha 410008, China; 2 International Collaborative Research Center for Medical Metabolomics, Xiangya Hospital Central South University, Changsha 410008, China

**Keywords:** inflammation, term labor, preterm labor, macrophages, myometrium, decidua, placenta

## Abstract

**Simple Summary:**

Inflammatory responses are critical in term and preterm labor initiation. The present study represents the inflammatory states in the mouse models’ key tissues associated with labor processes, such as placenta and uterine tissues decidua and myometrium, at the time of labor at term and preterm. We found that the infiltration of neutrophils and macrophages were increased in the above tissues at term labor and preterm labor. The expression of the factors such as cytokines and chemokines that are involved in inflammatory responses were also increased in these tissues. Moreover, macrophages exhibited proinflammatory states in these tissues at term and preterm labor. Our data suggest that macrophages shift to proinflammatory phenotypes at term and preterm labor, thereby contributing to the inflammation linked to term and preterm labor initiation.

**Abstract:**

Inflammation in gestational tissues plays critical role in parturition initiation. We sought to investigate the leukocyte infiltration and cytokine profile in uterine tissues to understand the inflammation during term and preterm labor in the mouse model. Preterm birth was induced by the administration of lipopolysaccharide (LPS) or RU38486. The populations of leukocytes were determined by flow cytometry. Macrophages were the largest population in the myometrium and decidua in late gestation. The macrophage population was significantly changed in the myometrium and decidua from late pregnancy to term labor and significantly changed at LPS- and RU386-induced preterm labor. Neutrophils, T cells, and NKT cells were increased in LPS- and RU38486-induced preterm labor. The above changes were accompanied by the increased expression of cytokines and chemokines. In late gestation, M2 macrophages were the predominant phenotype in gestational tissues. M1 macrophages significantly increased in these tissues at term and preterm labor. IL-6 and NLRP3 expression was significantly increased in macrophages at labor, supporting that macrophages exhibit proinflammatory phenotypes. NLRP3 inflammasome inhibitor MCC950 mainly suppressed macrophage infiltration in the myometrium at term labor and preterm labor. Our data suggest that the M1 polarization of macrophages contributes to inflammation linked to term and preterm labor initiation in gestational tissues.

## 1. Introduction

Preterm birth (PTB) is the leading cause of perinatal mortality worldwide. It is responsible for most newborn morbidity, including cerebral palsy, cognitive impairment, blindness, deafness, respiratory illness, and complications of neonatal intensive care [1]. The etiopathogenesis of PTB is associated with multiple factors, such as infection, uteroplacental ischemia, decidual hemorrhaging, defects in the mother’s immunological tolerance to the fetus, allergies, excessive uterine size, cervical incompetence, maternal and fetal stress, and hormonal abnormalities [2]. However, so far, it lacks effective interventions for PTB. Such failure is mainly attributed to the fact that the fundamental mechanisms underlying the initiation of physiological parturition are not fully understood. However, the parturition processes are similar in term and preterm birth, which are characterized by uterine changes, including cervical softening and ripening, the activation of amnion, chorion, and decidua, and the conversion of uterine smooth muscle from being in a quiescent state to a contractile state [3].

In recent years, an increasing body of evidence has indicated that inflammation is involved in each session of the parturition processes, i.e., cervical softening and ripening, the activation of fetal membranes, and the uterine transition to a contractile state [3,4,5]. Leucocytes are accumulated and the levels of inflammatory mediators are elevated in gestational tissues, including the cervical stroma, myometrium, decidua, and fetal membrane during spontaneous labor at term as well as preterm in pregnant women [6,7,8]. Pro-inflammatory cytokines initiate a cascade of inflammatory mediator production, including matrix metalloproteinases and prostaglandins, which in turn lead to cervical dilation, rupture of the membranes, and uterine contractions [9,10]. Intrauterine infection accounts for 25–40% of all premature human deliveries, which causes the early initiation of the inflammatory pathway [9,11,12]. Intrauterine infections can arise systemically or by commensal bacteria ascending from the female genital tract [3,9]. Several mouse models of preterm birth have therefore been developed [4]. Among them, the most used model is lipopolysaccharide (LPS)-induced preterm birth in mice. LPS can act on toll-like receptor 4 (TLR4) in various tissues, leading to inflammatory responses [5,13]. It is well known that progesterone is a key hormone in maintaining pregnancy in humans and rodents. Spontaneous labor is initiated by a series of biochemical events associated with progesterone withdrawal or functional withdrawal [6,14]. Thus, preterm birth induced by progesterone receptor antagonist RU38486 in rodents is a frequently used model for studying preterm birth. One of key mechanisms of progesterone maintenance of pregnancy is that it can inhibit NF-κB signaling, thereby suppressing the generation of inflammatory mediators and the expression of uterine activation proteins in the uterus. We recently showed that the inhibition of inflammation delays not only LPS-induced preterm birth, but also term and RU38486-induced preterm birth [15,16,17].

The distribution and phenotype of various immune cells can vary in the compartments of gestational tissues during gestational progress [18,19]. Different types of immune cells exert differential effects on pregnancy maintenance and labor initiation [7]. For instance, natural killer T (NKT) cells are involved in the pathophysiology of preterm labor associated with infection and other T cell subsets participate in LPS- and RU38486-induced preterm birth [20,21]. Neutrophils are proposed to play a central role in the cervical ripening process [22]. Macrophage accumulation is enhanced in the myometrium and decidua in LPS- and RU38486-induced preterm birth [18,23]. More recently, it has been reported that macrophages are critical for pregnancy maintenance, and macrophages’ depletion leads to preterm labor [24,25]. Pique-Regi et al. [15] showed that NK-cell and activated T cell signatures are increased with labor at term and macrophage, monocyte, and started T cell signatures with preterm labor in human placentas. It is known that macrophages in the tissues are polarized according to the environment, forming two main subtypes, M1 and M2 [23,26,27]. M1 macrophages display proinflammatory responses and produce pro-inflammatory-related factors such as IL-6 and tumor necrosis factor α(TNFα). In contrast, M2 macrophages exert anti-inflammatory responses. Some studies have implicated M1 macrophages accumulation in decidua before the onset of term labor in humans and rodents [28,29,30]. Gomez-Lopez et al.’s study has revealed that M2 decline in the maternal–fetal interface contributes to term birth [20]. The dynamic changes in phenotypes of macrophages in other gestational tissues, such as the placenta, at term labor and preterm birth require further investigation. Although the infiltration of some types of immune cells in the myometrium and decidua from the late gestational stage to term or preterm labor in animal models have been described by several studies [31,32,33], the dynamic changes in the main types of immune cells, including myeloid cells and lymphocytes in gestational tissues, remain largely unknown. Although inflammatory responses play a critical role in LPS-induced preterm birth and RU38486-induced preterm birth, it is interesting to understand the similarity and difference in inflammatory microenvironments within gestational tissues in these two preterm labor models.

The nucleotide-binding and oligomerization domain-like (Nod) receptor family pyrin domain-containing 3 (NLRP3) inflammasome is involved in various inflammatory responses [34]. Some studies have demonstrated that NLRP3 inflammasome is activated in gestational tissues in the term and preterm birth of humans and rodents [17,35]. We recently revealed that NLRP3 inflammasome inhibitor MCC950 prolongs the onset of term birth and postpones RU38486- and lipopolysaccharide (LPS)-induced preterm birth in the mouse model [17]. However, the effects of MCC950 treatments on the infiltration of immune cells in gestational tissues need to be investigated.

The purposes of the present study were to investigate the dynamic changes of immune microenvironments and the effects of NLRP3 inflammasome inhibitor on leucocyte infiltration in gestational tissues in late gestational stage to term labor and preterm labor in mouse models. We first observed the abundance of various immune cells, including neutrophils, macrophages, NK cells, NKT cells, and T cells in the uterus, decidua, and placenta in pregnant mice from late pregnancy to labor and before and after the onset of labor in the models of RU38486- and LPS-induced preterm birth. Secondly, we examined dynamic changes in M1 and M2 populations’ phenotypes and IL-6 and NLRP3 expression in the above tissues in term and preterm labor. Thirdly, we examined the immune cells in the gestational tissues in response to NLRP3 inflammasome inhibitor MCC950 treatment at term and preterm birth. As the immune cells are attracted to the site of inflammation by chemokines and cytokines and the inflammatory responses lead to increased cytokine levels, the expression levels of proinflammatory cytokines (IL-1β, IL-6, and TNFα) and chemokines (CXCL1, CXCL2, CCL2, and M-CSF) were examined in gestational tissues. Our data provide new evidence regarding inflammatory responses in gestational tissues linked to term and preterm labor initiation, which immediately gains deep insight into understanding the mechanisms responsible for term and preterm labor.

## 2. Materials and Methods

### 2.1. Animal Protocols

ICR mice (20–25 g body weight, 6–8 weeks old) were obtained from Shanghai SLAC Laboratory Animal Co (Shanghai, China) and then housed in social groups of 3–5 in a cage with regular light–dark cycles (lights on at 7:00 a.m., lights off at 7:00 p.m.) under controlled temperature (22 ± 2 °C) and humidity (50 ± 10%), and were given standard diet and water ad libitum. All animal procedures were carried out in accordance with the guidelines for the use of laboratory animals published by the People’s Republic of China Ministry of Health (May 2016), with the approval of the Ethics Board of Medical Research of Xiangya Hospital, Central South University. Breeding females were handled daily for 1 week. One to three virgin female mice were housed with a male mouse overnight beginning at 20:00. If female mice were found to have vaginal plugs at 8:00 the following day, this was designated to be gestational day (GD) 0.5. There were three experiments in the present study. In the first experiment, timed-pregnant mice at GD16.75, GD18, and labor (delivery of the first pup) were sacrificed by deep anaesthetization (urethane and alpha-chloralose, i.p.). Then, the uterus, decidua, and placentas were collected. Some of them were frozen immediately in liquid nitrogen and then stored at −80 °C. The rest of the tissues were put in D-Hank’s solution (4 °C) for the following flow cytometry. In the second experiment, timed-pregnant mice at GD16 were administered with RU38486 (Sigma-Aldrich, Burlington, VT, USA) at 7.5 mg/kg (s.c.), and timed-pregnant mice at GD16.25 were injected with Escherichia coli 0111:B4 LPS (Sigma-Aldrich) at 0.5 mg/kg (i.p.). The reagent of RU38486 was dissolved in absolute alcohol and then diluted in corn oil (Sigma-Aldrich). The corresponding controls were injected with the same volume of corn oil (s.c.) or saline (i.p.). The dosages of the above reagents were chosen based on our previous study [17]. At the time of labor, the mice were sacrificed by deep anaesthetization. Then, the uterus, decidua, and placentas were collected. In the third experiment, the timed mice were injected (i.p.) with NLRP3 inflammasome inhibitor MCC950 (Sigma-Aldrich) at 200 mg/kg (i.p.) in 100 μL sterile saline at 8:30 at GD18.5. They received a second injection 12 h later. Control timed pregnant mice received 100 μL saline (i.p.). At the time of labor, the mice were sacrificed by deep anaesthetization, and the uterus, decidua, and placentas were then collected. Timed mice (GD16) were injected with RU38486 (s.c.) and MCC950 at 200 mg/kg (i.p.) in the meantime. Twelve hours later, the mice were injected with the same dosage of MCC950. Corresponding control timed pregnant mice received an injection of the same volume of corn oil and saline. At the time of labor, the mice were sacrificed by deep anaesthetization, and the uterus, decidua, and placentas were collected. Timed mice (GD16.25) were injected with Escherichia coli 0111:B4 LPS at 0.5 mg/kg and MCC950 at 200 mg/kg (i.p.) in the meantime. The control mice received an injection of the same volume of saline. At the time of labor, the mice were sacrificed by deep anaesthetization, and the uterus, decidua, and placentas were collected.

### 2.2. Cell Isolation

The myometrial and decidua tissues were minced into small pieces and digested in 0.025 mg/mL DNase type I (Sigma, St. Louis, MO, USA) and 1 mg/mL Collagenase Type IV (Gibco, Thermo Fisher Scientific, Waltham, MA, USA) for 0.5 h at 37 °C during constant gentle shaking. The placental tissues were kept on PBS (Servicebio, Wuhan, China) and cut into small pieces. The collected cells were filtered through a 70 µm cell strainer (Biosharp, China). The fragments of tissue remained in the cell strainer and the cell suspension was passed through it. Isolated cells from the uterine, decidual, and placental tissues were washed twice with PBS (Servicebio, Wuhan, China) and centrifuged at 350× *g* at 4 °C for 5 min.

### 2.3. Monoclonal Antibodies (mAbs) and Reagents

Fluorescein-conjugated anti-mouse mAbs, including anti-CD45-BV711 (30-F11), anti-CD11b-BV510 (M1/70), anti-CD3-PE-Cy7 (145-2C11), anti-Ly6G-PerCP-Cy5.5 (1A8), and anti-NK1.1-BV421 (PK136), were purchased from BioLegend (BioLegend Inc., San Diego, CA, USA). Dye (Fixable viability 780)-APC-Cy7 and anti-F4/80-Alexa fluor 647 (T45-2342) were obtained from BD Pharmingen (BD Bioscience, Becton, NJ, USA). Anti-NLRP3-Alexa Fluor 488 (768319) was obtained from from R&D Systems, and anti-iNOS-PE-Cy7 (CXNFT), anti-Arg1-PE (A1exF5), and anti-IL-6-eFluor 450 (MP5-20F3) were obtained from Invitrogen (Invitrogen, ThermoFisher Ltd., Shanghai, China).

### 2.4. Flow Cytometry

As described previously, surface and intracellular staining was carried out by multicolor flow cytometry (FCM) [36]. For cell surface staining, single-cell suspensions from various organs were stained for 10 min with Fixable Viability Stain 780 Stock Solution at room temperature, protected from light. Without washing, the cells were incubated with TruStain FcX™ PLUS (anti-mouse CD16/32) Antibody at room temperature in the dark for 10 min to block Fc receptors. After washing, the cells were resuspended in 100 μL PBS with various fluorescein-conjugated mAbs at room temperature in the dark for 30 min. After incubation, the labeled cells were washed twice with PBS and collected. For the intracellular staining of iNOS, Arg1, IL-6, and NLRP3, after the cells were stained for surface markers, the cells were fixed and permeabilized with the Fixation/Permeabilization Buffer set for 45 min, followed by resuspension with permeabilization buffer and incubation with directly conjugated specific antibody for 45 min at room temperature. Then, they were washed twice with PBS and then tested. All samples were collected on a Cytek Athena flow cytometer (Cytek Biosciences, Fremont, CA, USA), and all sata were analyzed with the FlowJo 10.8.1 software (TreeStar, Woodburn, OR, USA).

### 2.5. Total RNA Extraction and Quantitative Real-Time PCR (Q-PCR)

TRIzol reagent (AGBIO Co. Ltd., Changsha, China) was used to extract the total RNAs from the frozen tissues following the company’s instructions. Then, 1 µg RNA was reverse transcribed to generate cDNA by using PrimeScript RT Master Mix Kit (TaKaRa Bio. Inc., Dalian, China). The primers used for Q-PCR were synthesized by Tsingke Biotechnology (Beijing, China). They are listed in Table 1. Q-PCR reaction was carried out on MiniOpticon™ Real-Time PCR Detection System (BioRad, Hercules, CA, USA). The reaction solution consisted of 2.0 μL diluted cDNA, 0.2 μM of each paired primer, and 1×ChamQ Universal SYBR qPCR Master Mix (Vazyme Biotechnology, Nanjing, China). The housekeeping gene β-actin was used as an internal control. The specificity of the PCR products was examined by a melting curve at the end of the amplification and subsequent sequencing. To determine the relative quantitation of gene expression for both the target and housekeeping genes, the comparative Ct (threshold cycle) method with arithmetic formulae (2^−ΔΔCt^) was used.

### 2.6. Statistical Analysis

Statistical analyses were performed using IBM SPSS Statistics 20 (IBM, Armonk, NY, USA). Normal distribution was assessed by Shapiro–Wilk test. Statistical significance was determined according to sample distribution and homogeneity of variance. Statistical comparisons between two groups were determined by a two-tailed Student’s *t* test. One-way ANOVA followed by Bonferroni’s post hoc test was performed for comparisons among multiple groups. *p* < 0.05 was considered statistically significant. All of the data are expressed as mean ± SEM, * *p* < 0.05, ** *p* < 0.01, *** *p* < 0.001, **** *p* < 0.0001.

## 3. Results

### 3.1. Dynamic Changes in Leukocyte Infiltration and Cytokine and Chemokine Expression in Myometrium, Decidua, and Placenta at Late Gestation and Labor

**Figure 1:**
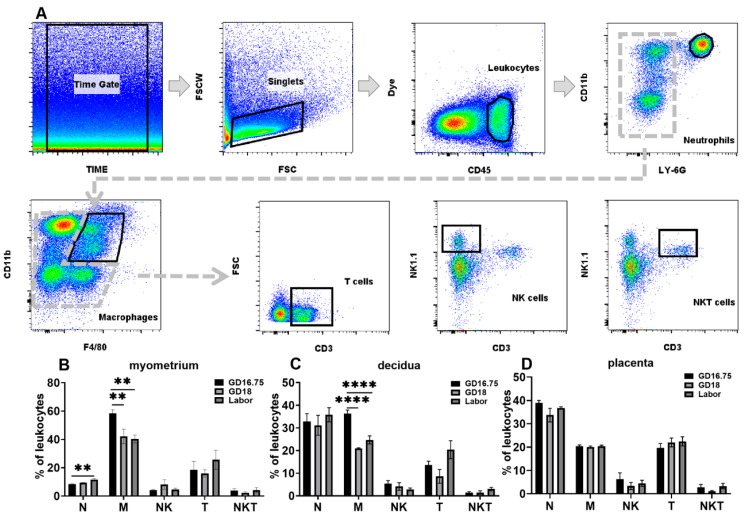
Dynamic changes of leukocyte subsets in myometrium, decidua, and placenta at late gestation and labor. The pregnant mice were sacrificed at GD16.75, GD18, and labor. Myometrium, decidua, and placenta were obtained for examination of the populations of neutrophils, macrophages, NK, T cells, and NKT cells by flow cytometry analysis. (**A**) Gating strategy used to identify neutrophils (Dye^−^CD45^+^CD11b^+^Ly6G^+^), macrophages (Dye^−^CD45^+^CD11b^+^F4/80^+^), T cells (Dye^−^CD45^+^CD11b^−^CD3^+^), NK cells (Dye^−^CD45^+^CD11b^−^CD3^−^NK1.1^+^), and NKT cells (Dye^−^CD45^+^CD11b^−^CD3^+^NK1.1^+^). (**B**) Populations of leukocyte subsets in myometrium; (**C**) populations of leukocyte subsets in decidua; (**D**) populations of leukocyte subsets in placenta. Data are expressed as mean ± SEM. n = 6 in each group. ** *p* < 0.01, **** *p* < 0.0001. N, neutrophils; M, macrophages.

To investigate dynamic changes in the various leukocyte populations in the myometrium, decidua, and placenta from late gestation to labor, flow cytometry analysis was applied to examine the infiltration of leukocytes in the above tissues. The populations of neutrophils, macrophages, NK cells, T cells, and NKT cells were determined in pregnant mice at GD16.75, GD18, and labor. The gating strategy is shown in Figure 1A.

In the myometrium, macrophages were the largest population of immune cells in the late gestational phase representing 58.48% ± 6.78 of the leucocytes at GD16.75. The second largest population of leucocytes is T cells, which represented 18.47% ± 13.42 of the leucocytes. Neutrophils and NK cells represented 8.44% ± 0.88 and 4.28% ± 1.46 of the leukocytes, respectively. Neutrophil and macrophage infiltration were significantly changed from GD16.75 to labor (Figure 1B). The number of neutrophils was significantly increased at the onset of labor compared with GD16.75, while it was not significantly changed at GD18 compared with GD16.75. Macrophage infiltration was seriously decreased at GD18 compared with GD16.75 and remained at a low level at labor. The populations of other immune cells, including NK cells, T cells, and NKT cells, were not significantly changed (Figure 1B).

In the decidua, the largest population of immune cells was neutrophils and macrophages, 32.81% ± 9.89 and 36.26% ± 4.61 of the leukocytes, respectively. Neutrophil infiltration was not significantly changed from GD16.75 to labor (Figure 1C). The numbers of macrophages were reduced considerably at GD18 compared with GD16.75 and remained at this level until the onset of labor (Figure 1C). Other immune cell infiltrations including NK cells, T cells, and NKT cells were not significantly different from GD16.75 to labor (Figure 1C).

In the placenta, the largest population of leukocytes was neutrophils, accounting for 38.89% ± 3.261 of the leukocytes. Macrophages and T cells were the second largest subsets of leucocytes at 20.31% ± 1.807 and 19.66% ± 5.462 of the leukocytes, respectively. There were no significant changes in neutrophils, macrophages, NK, T cells, and NKT cells at GD16.75, GD18, and labor (Figure 1D).

**Figure 2:**
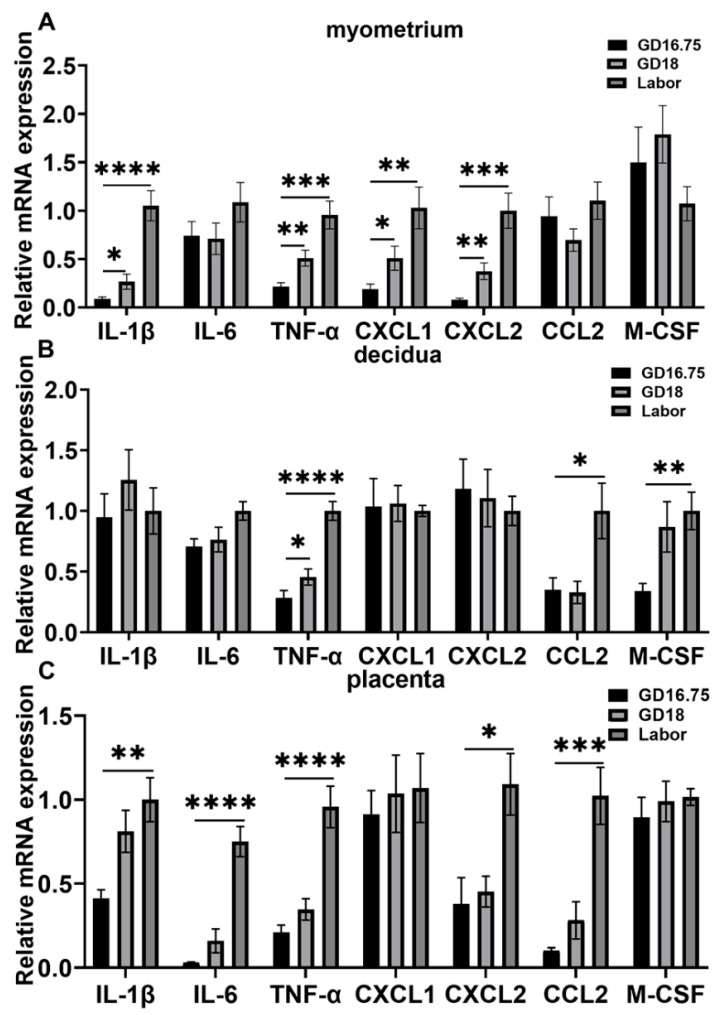
Dynamic changes of cytokine and chemokine expression in myometrium, decidua, and placenta from late gestation to labor. The pregnant mice were sacrificed at GD16.75, GD18, and labor. Myometrium, decidua, and placenta were obtained for determination of the mRNA levels of IL-1β, IL-6, TNFα, CXCL1, CXCL2, CCL2, and M-GSF by Q-PCR analysis. (**A**) Cytokine and chemokine mRNA levels in myometrium; (**B**) cytokine and chemokine mRNA levels in decidua; (**C**) cytokine and chemokine mRNA levels in placenta. Data are expressed as mean ± SEM. n = 6 in each group. * *p* < 0.05, ** *p* < 0.01, *** *p* < 0.001, **** *p* < 0.0001.

We then examined the expression levels of some cytokines and chemokines associated with the initiation of parturition and the accumulation of neutrophils and macrophages in the decidua and myometrium. Prior studies have shown that increased levels of IL-1β, IL-6, and TNFα are associated with the onset of labor [3,7,10,31]. CXCL1 and CXCL2 are the main chemokines of neutrophils, while CCL2 and M-GSF are chemokines of macrophages [37,38,39]. In the myometrium, the IL-1β and TNFα mRNA levels were significantly increased at GD18 compared with GD16.75, and further increased at labor in the myometrium (Figure 2A). The CXCL1 and CXCL2 mRNA levels were also increased at GD18 and further increased at labor compared with GD16.75. However, the IL-6, CCL2, and M-GSF levels were not significantly changed from GD16.75 to labor. In the decidua, the TNFα mRNA levels were increased at GD18 and further increased at labor (Figure 2B). The CCL2 and M-CSF levels were significantly increased at labor. The IL-1β, IL-6, CXCL1, and CXCL2 levels were not significantly changed from GD16.75 to labor. In the placenta, the IL-1β, IL-6, TNFα, CXCL2, and CCL2 mRNA levels were significantly increased from GD16.75 to labor (Figure 2C).

### 3.2. Macrophages Exhibit M1 Polarization and IL-6 and NLRP3 Expression Is Increased in Myometrium, Decidua, and Placenta at Labor

**Figure 3:**
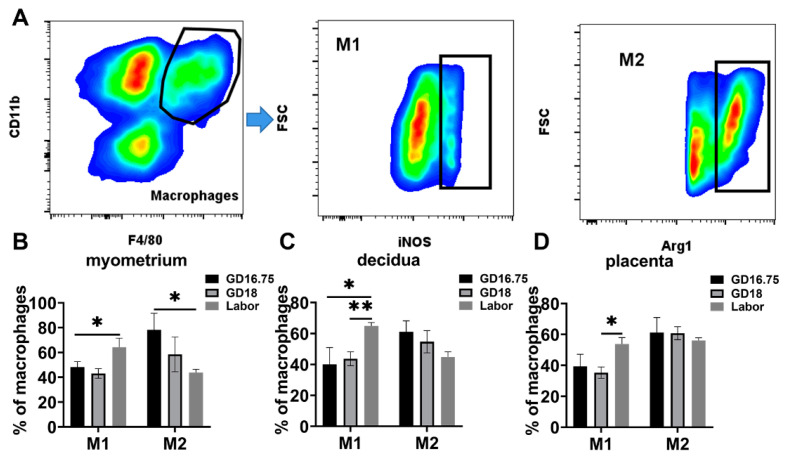
Characteristics in M1 and M2 populations in gestational tissues at late gestation and labor. The pregnant mice were sacrificed at GD16.75, GD18, and labor. (**A**) Gating strategy was used to identify M1 (Dye^−^CD45^+^CD11b^+^F4/80^+^iNOS^+^) and M2 (Dye^−^CD45^+^CD11b^+^F4/80^+^Arg1^+^). (**B**) Populations of M1 and M2 in myometrium; (**C**) populations of M1 and M2 in decidua; (**D**) populations of M1 and M2 in placenta. Data are expressed as mean ± SEM. n = 5 in each group. * *p* < 0.05, ** *p* < 0.01.

It is known that macrophages have M1 and M2 phenotypes. M1 displays a proinflammatory phenotype, while M2 displays anti-inflammatory states [23,26]. We then examined the phenotype of macrophages at late gestation and labor. In the myometrium, the population of M2 macrophages was higher than M1 types at GD16.75. The M1 phenotype macrophages were significantly increased at labor compared with GD16.75 and GD18. The M2 phenotype macrophages significantly declined at labor compared with GD16.75 (Figure 3A). In the decidua, the M2 population was also higher than the M1 population at GD16.75. The M1 phenotype macrophages were significantly increased at labor compared with GD16.75 and GD18. The M2 phenotype macrophages declined at labor compared with GD16.75 with no statistical significance (Figure 3B). The M2 population was also higher than the M1 population at GD16.75. Similarly, the M1 macrophages were significantly increased at labor compared with GD18 in the placenta. There was no significant difference in the M2 population from GD16.75 to labor (Figure 3C).

**Figure 4:**
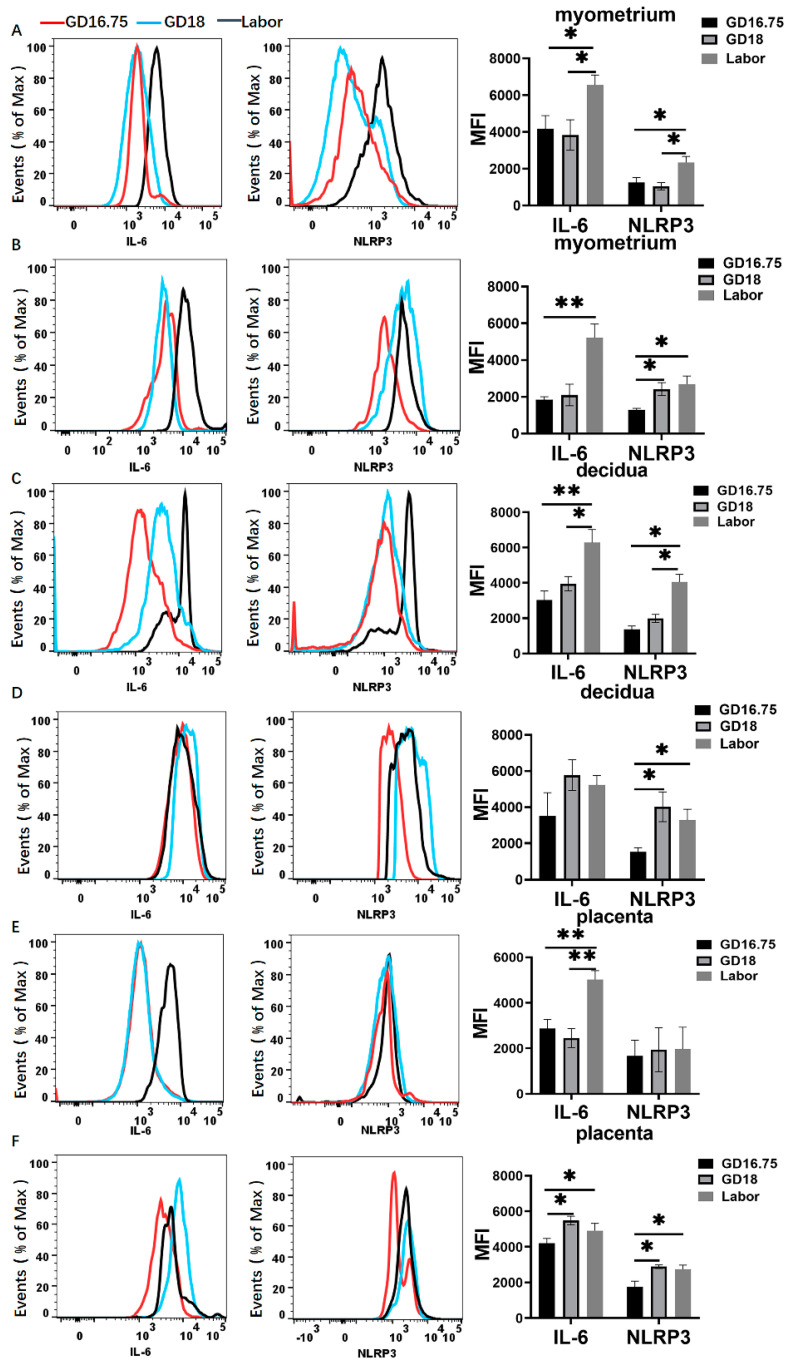
Characteristics in IL-6 and NLRP3 expression in gestational tissues at late gestation and labor. The pregnant mice were sacrificed at GD16.75, GD18, and labor. Myometrium, decidua, and placenta were obtained for determination of the expressions of IL-6 and NLRP3 by flow cytometry analysis. (**A**,**B**) Flow cytometry of IL-6 and NLRP3 expression among total leukocytes (**A**) or macrophages (**B**) in myometrium. (**C**,**D**) Flow cytometry of IL-6 and NLRP3 expression among total leukocytes (**C**) or macrophages (**D**) in decidua. (**E**,**F**) Flow cytometry of IL-6 and NLRP3 expression among total leukocytes (**E**) or macrophages (**F**) in placenta. Representative flow cytometry evaluating IL-6 and NLRP3 expressions (the left panel) and statistics calculated by the mean fluorescence intensity (MFI) of IL-6 and NLRP3 (the right panel) from total leukocytes (CD45^+^) or macrophages (CD11b^+^F4/80^+^). Data are expressed as mean ± SEM. n = 5 in each group. * *p* < 0.05, ** *p* < 0.01.

Prior studies have shown that IL-6 plays a critical role in the initiation of parturition in mice [40]. Moreover, we have previously demonstrated that NLRP3 inflammasome is involved in initiating term and preterm birth [17]. We therefore examined IL-6 and NLRP3 expression from GD16.75 to labor. In the myometrium, the expression of IL-6 and NLRP3 among the total leukocytes and macrophages was robustly increased at labor compared with GD16.75 (Figure 4A,B). The NLRP3 expression among the macrophages was significantly increased at GD18 and further increased at labor. In the decidua, the IL-6 and NLRP3 expression was significantly increased in the total leukocytes at labor compared to GD16.75 (Figure 4C). Among the macrophages, the NLRP3 expression was increased at GD18 and labor compared with GD16.75 (Figure 4D). In the placentas, the expressions of IL-6 and NLRP3 among the total leukocytes were significantly increased at labor compared with GD16.75 (Figure 4E). Among the macrophages, the IL-6 and NLRP3 expression was also significantly increased at GD18 and labor compared with GD16.75 (Figure 4F).

### 3.3. Changes in Leukocyte Subsets and Cytokine and Chemokine Expression in Myometrium, Decidua, and Placenta in LPS and RU38486 Preterm Birth

**Figure 5:**
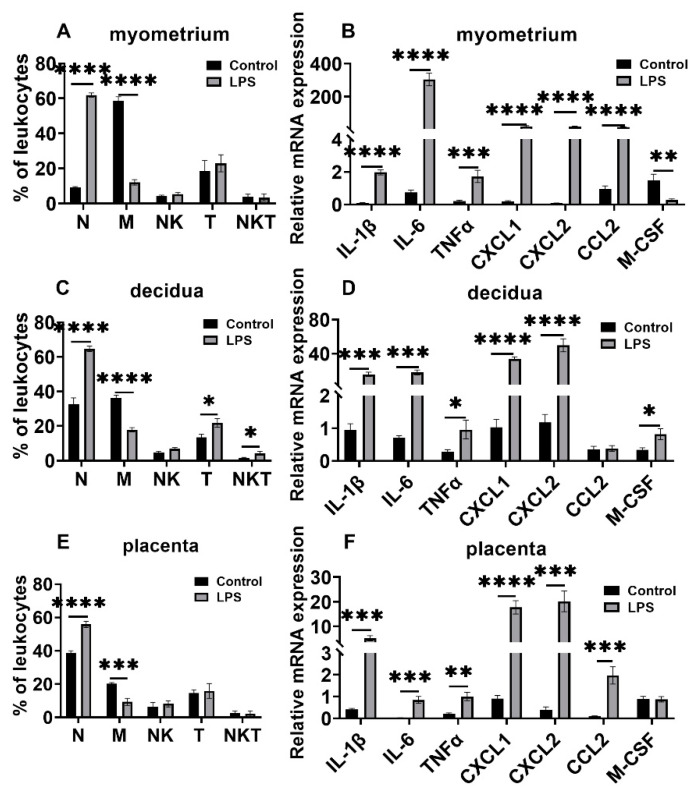
Changes in leukocyte subsets as well as cytokine and chemokine expression in gestational tissues at preterm labor induced by LPS. Pregnant mice were administered LPS or vehicle at GD16.25. The mice with LPS were sacrificed after the onset of labor. The control mice were sacrificed at the corresponding time of the LPS group. Myometrium, decidua, and placenta were obtained for determination of the populations of neutrophils, macrophages, NK, T cells, and NKT cells by flow cytometry analysis and the chemokine and cytokine expression by Q-PCR. (**A**,**B**) Leukocyte subsets and cytokine and chemokine mRNA levels in myometrium; (**C**,**D**) leukocyte subsets and cytokine and chemokine mRNA levels in decidua; (**E**,**F**) leukocyte subsets and cytokine and chemokine mRNA levels in placenta. Data are expressed as mean ± SEM. n = 6 in each group. * *p* < 0.05, ** *p* < 0.01, *** *p* < 0.001, **** *p* < 0.0001. N, neutrophils; M, macrophages.

As expected, LPS administration caused preterm labor in 10.36 ± 0.68 h after injection. In this model, the macrophage population was significantly decreased, whereas the neutrophils were robustly increased in the myometrium at preterm labor compared with the control. The population of T cells and NKT cells was not significantly changed at labor in response to LPS treatment (Figure 5A). The mRNA levels of IL-1β, IL-6, TNFα, CXCL1, CXCL2, and CCL2 were significantly enhanced, whereas the M-CSF level was decreased in the myometrium compared with the control (Figure 5B). In the decidua, the changed pattern in the neutrophil and macrophage populations was similar to that in the myometrium, i.e., the number of neutrophils significantly increased, whereas the macrophage population decreased considerably. T cell infiltration and NKT cell population were also significantly increased (Figure 5C). The IL-1β, IL-6, TNFα, CXCL1, CXCL2, and M-CSF expression levels significantly increased in the decidua in response to LPS treatment (Figure 5D). Like the changes in the myometrium, the neutrophils were increased while the macrophages were decreased in the placentas at preterm labor compared with the controls, and other immune cells were not significantly changed compared to the controls (Figure 5E). The IL-1β, IL-6, TNFα, CXCL1, CXCL2, and CCL2 expression levels were robustly increased in the placentas (Figure 5F).

**Figure 6:**
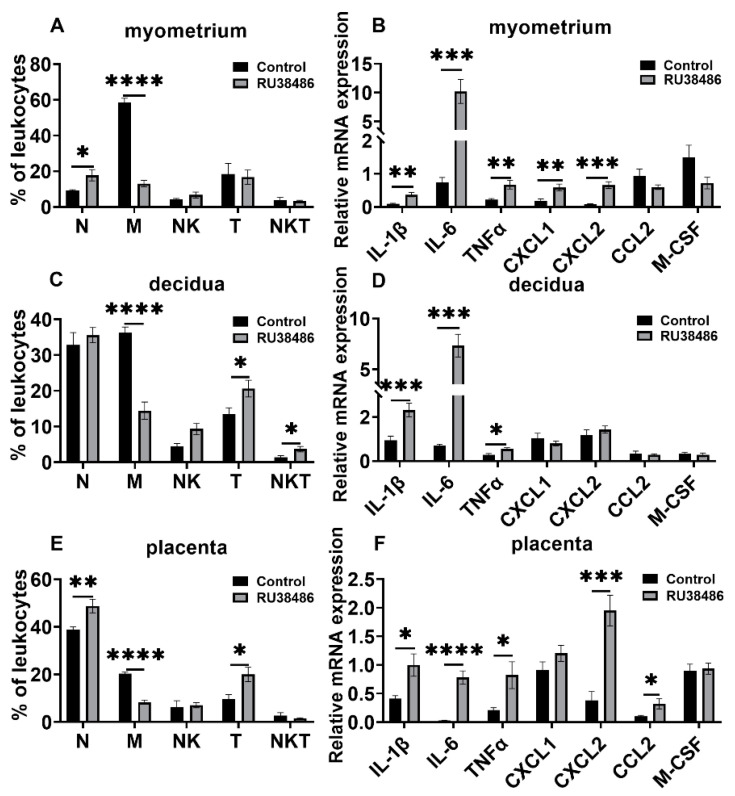
Changes in leukocyte subsets and cytokine and chemokine expression in gestational tissues at preterm labor induced by RU38486. Pregnant mice were administered RU38486 or vehicle at GD16. The mice with RU38486 were sacrificed after the onset of labor. The control mice were sacrificed at the corresponding time of the RU38486 group. Myometrium, decidua, and placenta were obtained for determination of leukocyte subsets and chemokine and cytokine expression. (**A**,**B**) Leukocyte subsets and cytokine and chemokine mRNA levels in myometrium; (**C**,**D**) leukocyte subsets and cytokine and chemokine mRNA levels in decidua; (**E**,**F**) leukocyte subsets and cytokine and chemokine mRNA levels in placenta. Data are expressed as mean ± SEM. n = 6 in each group. * *p* < 0.05, ** *p* <0.01, *** *p* < 0.001, **** *p* < 0.0001. N, neutrophils; M, macrophages.

RU38486 administration resulted in preterm labor in 16.61 ± 0.064 h after injection. In this preterm birth model, the macrophage population was significantly decreased in the myometrium, decidua, and placentas at preterm labor, while neutrophils were increased in the myometrium and placentas, but not the decidua. The T cell population increased dramatically in the decidua and placentas, but not in the myometrium at preterm labor. NKT cells were increased in decidua (Figure 6A,C,E). The IL-1β, IL-6, and TNFα mRNA levels were significantly increased in the myometrium, decidua, and placentas. The CXCL1 and CXCL2 mRNA levels were significantly increased in the myometrium, and the CXCL2 and CCL2 levels were significantly increased in the placentas compared with the controls. CCL2 and M-CSF were not considerably changed in the myometrium and decidua, and CXCL1 and M-CSF were not significantly changed in the placentas (Figure 6B,D,F).

### 3.4. Characteristics in M1 and M2 Populations and IL-6 and NLRP3 Expressions in Myometrium, Decidua, and Placenta at Preterm Labor Induced by LPS and RU38486

**Figure 7:**
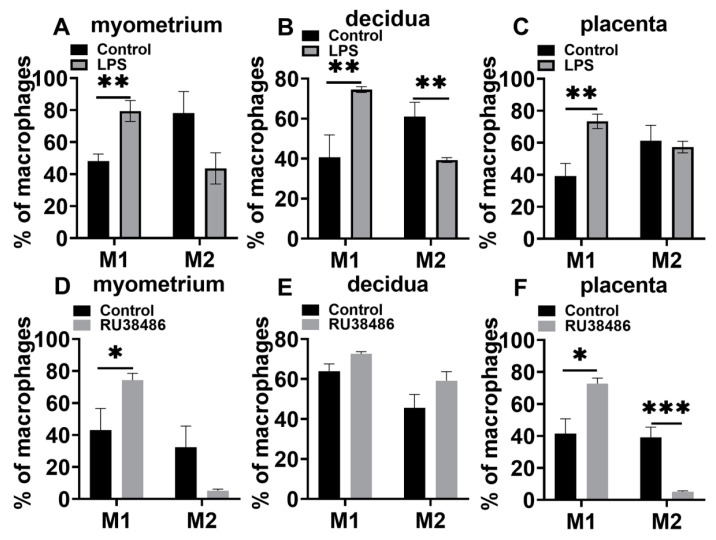
M1 and M2 populations in gestational tissues at preterm labor induced by LPS and RU38486. Pregnant mice were administered LPS (**A**–**C**) at GD16.25 or RU38486 (**D**–**F**) at GD16. The mice with LPS or RU38486 were sacrificed after the onset of labor. The control mice were administered the same volume of saline and sacrificed at the corresponding time of LPS or RU38486 group. (**A**,**D**) Populations of M1 and M2 in myometrium; (**B**,**E**) populations of M1 and M2 in decidua; (**C**,**F**) populations of M1 and M2 in placentas. Dye^−^CD45^+^CD11b^+^F4/80^+^iNOS^+^ cells were considered as M1, while Dye^−^CD45^+^CD11b^+^F4/80^+^Arg1^+^ cells were considered as M2. Data are expressed as mean ± SEM. n = 5 in each group. * *p* < 0.05, ** *p* < 0.01, *** *p* < 0.001.

In the LPS-induced preterm birth model, the M1 population was significantly increased in the myometrium, decidua, and placentas during labor compared with the controls. The M2 population declined in the myometrium and decidua compared with the controls, although there was no statistical significance in the myometrium (Figure 7A–C). In the RU38486-induced preterm birth model (Figure 7D–F), the M1 populations were significantly increased in the myometrium and placenta and slightly increased in the decidua. The M2 populations were decreased in the myometrium (without statistical significance) and placenta.

**Figure 8:**
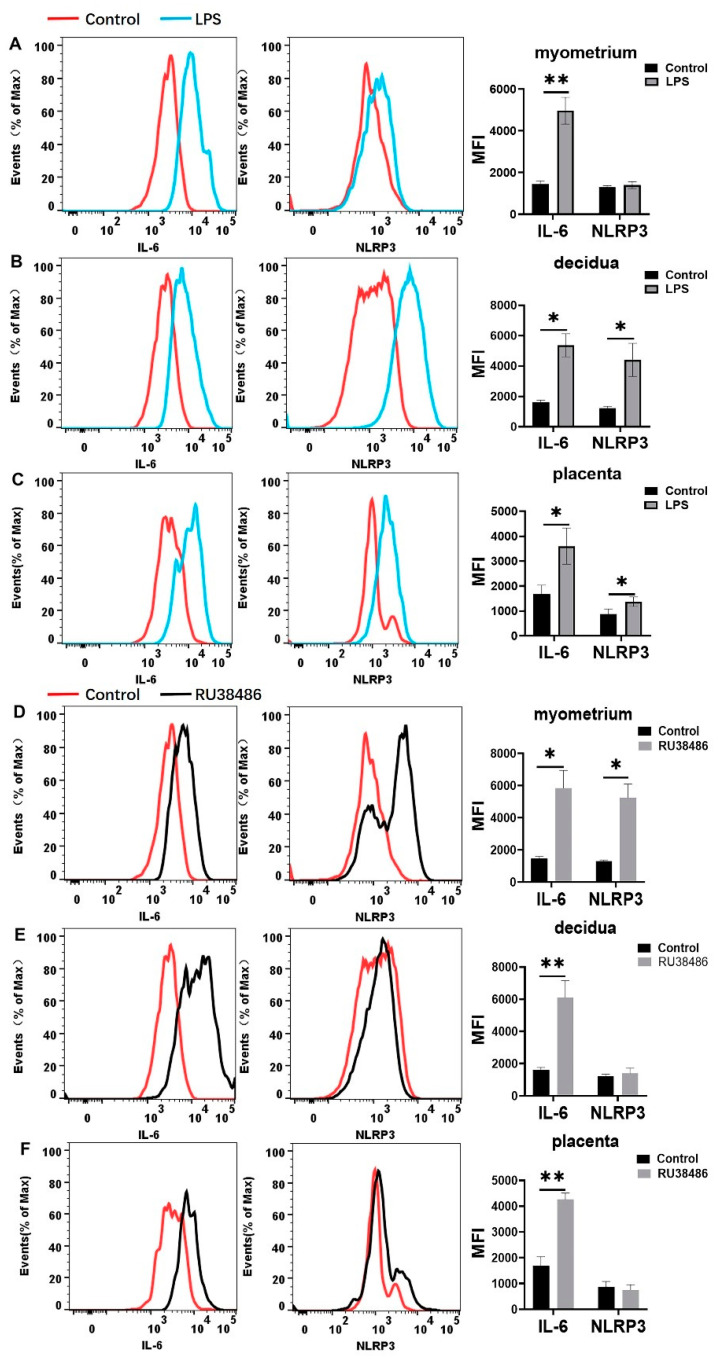
IL-6 and NLRP3 expression in gestational tissues at preterm labor induced by LPS and RU38486. Pregnant mice were administered LPS (**A**–**C**) at GD16.25 or RU38486 (**D**–**F**) at GD16. The mice with LPS or RU38486 were sacrificed after the onset of labor. The control mice were administered the same volume of saline and sacrificed at the corresponding time of LPS or RU38486 group. (**A**,**D**) Flow cytometry of IL-6 and NLRP3 expression in myometrium. (**B**,**E**) Flow cytometry of IL-6 and NLRP3 expression in decidua. (**C**,**F**) Flow cytometry of IL-6 and NLRP3 expressions in placenta. Representative flow cytometry evaluating IL-6 and NLRP3 expression (the left panel) and statistics calculated by the MFI of IL-6 and NLRP3 (the right panel) from macrophages (CD11b+F4/80+) in myometrium or decidua or placentas. Data are expressed as mean ± SEM. n = 5 in each group. * *p* < 0.05, ** *p* < 0.01.

The expression of IL-6 and NLRP3 among macrophages was further analyzed. As shown in Figure 8A–C, the IL-6 expression among the macrophages was significantly increased in the myometrium, decidua, and placentas. In contrast, NLRP3 expression was significantly increased in the decidua and placentas in LPS-induced preterm labor. In the RU38486-induced model (Figure 8D–F), the IL-6 expression among the macrophages was also considerably increased in the myometrium, decidua, and placentas. The NLRP3 expression was significantly increased in the myometrium, although it was slightly increased in the decidua and placentas.

### 3.5. Effects of MCC950 Treatment on Leukocyte Subsets in Gestational Tissues at Term and Preterm Labor

We have previously shown that MCC950, an inhibitor of NLRP3 inflammasome, prolongs term birth and LPS- and RU38486-induced preterm birth in mice [17]. We therefore examined whether the leukocyte populations are affected by MCC950 treatment at term and preterm labor.

**Figure 9:**
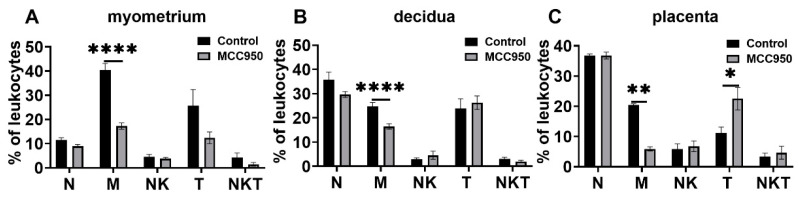
Effects of MCC950 treatment on leukocyte subsets in gestational tissues at term labor. Pregnant mice were administered MCC950 (i.p.) at GD18.5. The mice with MCC950 treatment were sacrificed at the onset of labor. The control mice were administered the same volume of saline and sacrificed at the onset of labor. Myometrium, decidua, and placentas were obtained for determination of neutrophils, macrophages, NK, T cells, and NKT cells by flow cytometry analysis. (**A**) Populations of leukocyte subsets in myometrium; (**B**) populations of leukocyte subsets in decidua; (**C**) populations of leukocyte subsets in placenta. Data are expressed as mean ± SEM. n = 6 in each group. * *p* < 0.05, ** *p* <0.01, **** *p* < 0.0001. N, neutrophils; M, macrophages.

MCC950 administration at GD18 could significantly postpone parturition time [17]. At labor, the main changes in leukocyte populations were macrophages in the myometrium, decidua, and placentas in response to MC950 treatment. It was significantly decreased in these tissues in the MCC950 group compared with the control group. T cells were increased in the placentas in response to MCC950 treatment compared with the vehicle control (Figure 9). Other immune cells were not significantly changed in response to MCC950 treatment. We also examined NLRP3 inflammasome activation upon MCC950 treatment.

**Figure 10:**
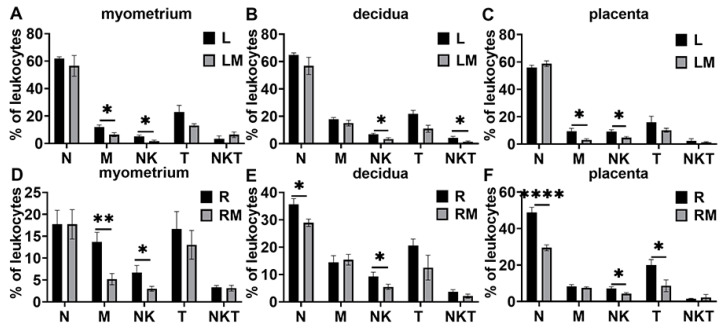
Effects of MCC950 treatment on leukocyte subsets in gestational tissues at the time of labor in LPS- and RU38486-induced preterm birth. Pregnant mice were administered LPS alone or LPS plus MCC950 at GD16.25 (**A**–**C**) or with RU38486 alone or RU38486 with MCC950 (**D**–**F**) at GD16. The mice were sacrificed after the onset of labor. Myometrium, decidua, and placentas were obtained for determination of neutrophils, macrophages, NK, T cells, and NKT cells by flow cytometry analysis. (**A**,**D**) Populations of leukocyte subsets in myometrium; (**B**,**E**) populations of leukocyte subsets in decidua; (**C**,**F**) populations of leukocyte subsets in placenta. Data are expressed as mean ± SEM. n = 6 in each group. * *p* < 0.05, ** *p* < 0.01, **** *p* < 0.0001. L, LPS; LM, LPS + MCC950; R, RU38486; RM, RU38486 + MCC950; N, neutrophils; M, macrophages.

In the LPS-induced preterm birth model (Figure 10A–C), MCC950 treatment decreased macrophage infiltration in the myometrium and placenta at the time of preterm labor. In addition, MCC950 treatment suppressed NKT cells in decidua at preterm labor. It also inhibited NK cells in the myometrium, decidua, and placentas.

In the RU38486-induced preterm birth model (Figure 10D–F), MCC950 inhibited macrophage populations in the myometrium, and neutrophil populations in the decidua and placentas at preterm labor. It also suppressed NK cells in the myometrium, decidua, and placentas during preterm labor.

## 4. Discussion

In the present study, we systemically studied the populations of various leukocyte types within the myometrium, decidua, and placentas at term labor and LPS- and RU38486-induced preterm labor in pregnant mice. It was found that the macrophage population was the largest population and significantly changed in the myometrium and decidua at term labor. The macrophage population was also affected by LPS treatment and RU38486 treatment in the myometrium and decidua, suggesting that macrophages are involved in initiating labor in term and preterm labor in these tissues. An increased population of neutrophils occurred in the myometrium at term labor and LPS- and RU38486-induced preterm labor, suggesting that this leukocyte subset might contribute to inflammation in the myometrium. Of note, T cells and NKT cells were increased in the decidua at labor in LPS- and RU38486-induced preterm birth models, but not term birth models, suggesting that these cells might be involved in initiating preterm birth.

It is well recognized that neutrophils are one of the major players during acute inflammation, and are the first leukocyte subset to be recruited to an inflammatory site [37,41]. Many studies have shown that neutrophils are recruited in gestational tissues in term and preterm labor in human and rodent models. For instance, some studies have shown that neutrophils are infiltrated in the cervix and myometrium of women with term labor compared with pregnant women at term without labor [7]. The increased flux of neutrophils is found in decidua at preterm labor in pregnant women with chorioamnionitis [42]. In mouse models, it has been reported that the influx of neutrophils is increased in the decidua and myometrium at term labor and LPS-induced preterm labor, but not RU38486-induced preterm labor [24,31,43]. In the present study, we showed that neutrophils were increased in the myometrium at term birth and LPS- and RU38486-induced preterm birth. In the decidua, neutrophil influx was increased at LPS-induced preterm labor, but not term labor and RU38486-induced preterm birth. These data were generally consistent with the studies by Shynlova et al. [24,31], but some differences, such as neutrophil infiltration in the myometrium, are noted, which might be attributed to the differences in the collecting time of the samples between the two groups. Of note, increased numbers of neutrophils in the placentas were found in LPS- and RU38486-induced preterm labor, but not term labor, suggesting that the recruitment of neutrophils contributes to the inflammation in the placenta in preterm birth. Given that there was a three- to tenfold increase in neutrophils in the decidua and myometrium in LPS-induced labor, this implies that neutrophils are involved in the process of infection-induced preterm labor. In fact, several studies have reported that a large influx of neutrophils invades the decidua and myometrium in LPS-induced preterm labor [44]. However, it has been reported that the depletion of neutrophils by neutralized antibodies does not prevent LPS-induced preterm birth and does not affect the timing of term labor [7,43]. Thus, it seems that neutrophils are not the key player in term labor and infection-induced preterm labor. However, since neutrophils secrete inflammatory mediators and matrix metalloproteinases (MMPs) [45,46], their contribution to the inflammation within gestational tissues in infection-associated preterm birth cannot be excluded.

It has been implicated that macrophages contribute to term and preterm labor [25,47]. Macrophages are the largest population in the myometrium and decidua in late gestation in rodents [24,25]. Some studies have shown that the number of macrophages in the uterus is increased at GD15 compared with nonpregnant controls, and then declines one day before birth in pregnant mice [28,48]. In rats, it is also found that uterine macrophages are decreased before labor [28]. Shynlova et al. [31] have shown that macrophage infiltration has a downward trend in the myometrium at term labor in mice. Our study also showed that the number of macrophages in the decidua and myometrium decreased at GD18. Moreover, we found that macrophages were significantly decreased in the myometrium, decidua, and placentas in LPS- and RU38486-induced preterm labor. More recently, Gomez-Lopez et al. [25] reported that the depletion of maternal macrophages leads to preterm birth. Collectively, this indicates that the decreased infiltration of macrophages in the myometrium and decidua contributes to the initiation of labor. We previously showed that MCC950 could prolong normal labor and LPS- and RU38486-induced preterm labor [17]. Of note, MCC950 mainly affected the number of macrophages in the myometrium and decidua in term labor and preterm labor [49,50]. In the mice with MCC950 treatment, the number of macrophages was further decreased in the myometrium at labor compared with the corresponding controls. This suggests that the initiation of labor is associated with decreased macrophages, even if it is delayed.

It is known that the function of macrophages is associated with the phenotypes of macrophages, i.e., M1 displays a proinflammatory state and M2 exhibits an anti-inflammatory function [23,26,27]. We found that the M2 phenotype was the predominant phenotype of the macrophages in late gestation, and M1 macrophages were significantly increased at term labor in the myometrium and decidua and became the predominant phenotype, although the number of macrophages was decreased in these tissues. The M1 population was significantly increased in the placentas at labor, although the total macrophage population was not changed from late gestation to labor. M1 polarization also occurred in gestational tissues in LPS- and RU38486-induced preterm labor. Given that the gestational tissues exhibit a pro-inflammatory state at term and preterm labor [3,51], such changes in the macrophage phenotype facilitate an inflammatory state in gestational tissues. It has been demonstrated that NLRP3 inflammasome contributes to M1 polarization, and M1 macrophages tend to secrete proinflammatory cytokines, such as IL-6 [23,26,52]. We also found that the expression of NLRP3 and IL-6 in macrophages was significantly increased in gestational tissues at term and preterm labor. It is known that the functions of macrophages are associated with the mediators released. In fact, M2 can be divided into subtypes M2a, M2b, M2c, and M2d [23]. The subtypes of M2 macrophages can exert divergent functions as the factors expressed differ. For instance, M2b macrophages can express TNFα, while M2c can express TGFβ, although both express IL-10. Thus, it would be important to investigate the dynamic profile of chemokines and cytokines in macrophages in different compartments of gestational tissues during pregnancy.

In the present study, we found that the population of T cells and NKT cells was significantly increased in decidua in LPS- and RU38486-induced preterm labor. Some studies have demonstrated that decidual NKT cells are involved in LPS-induced preterm labor in mice [20]. Recently, the studies of the Gomez-Lopez group have shown that effector memory and activated maternal T cells in the decidua lead to pathological inflammation, thereby contributing to preterm labor [21], and reduced regulatory T cells (Tregs) at the maternal–fetal interface are also associated with idiopathic preterm birth [53]. Some studies have shown that the B regulatory (Breg) cells are decreased in number and are functionally impaired in the maternal circulation of women with preterm birth [54], and B cells in cord blood secrete more proinflammatory cytokines in preterm birth babies than term birth babies [55], suggesting that B cells are also involved in preterm birth. More details of T and B cell information such as subtypes and profiles of cytokines in gestational tissues at term birth and preterm birth should be investigated in our future studies.

In addition, we found that the infiltration of neutrophils and macrophages was changed in the placenta in LPS- and RU38486-induced preterm labor. Since the placenta belongs to fetal tissues, the above changes might reflect the changes in immune cells invaded by the fetus. More recently, Sheller-Miller et al. [56] have demonstrated that LPS causes the increased infiltration of neutrophils in the placentas of pregnant mice. Thus, it suggests that fetal and maternal responses are involved in preterm labor.

Proinflammatory cytokines and chemokines including IL-1β, IL-6, TNFα, and CCL2 are significantly increased in human gestational tissues during term and preterm labor [10,32]. In fact, these cytokines can be secreted by myometrial cells [57]. It has been shown that IL-1β, IL-6, and TNFα can induce the expression of uterine-activated proteins (COX-2, connexin 43, and oxytocin receptor) in the human myometrium [7,10,32], which is the key event for the uterine activation of contractions. In animal studies, the administration of IL-1β can induce preterm labor, and IL-1 receptor antagonist inhibits LPS-induced preterm labor [58], while IL-6 gene deficiency leads to delayed term labor [59]. In the present study, we found that IL-1β, IL-6, TNFα, and CCL2 expression significantly increased in the myometrium at term labor and LPS- and RU38486-induced preterm labor, respectively, supporting that these cytokines are involved in the process of labor in the myometrium. We also found that the expression of these cytokines was also increased in the decidua and placenta at LPS- and RU38486-induced preterm labor, which contributes to the inflammatory state in these tissues in preterm birth. Many cytokines contribute to M1 and M2 polarization [23,26,27]. For instance, TNFα, interferon γ, and GM-CSF induce M1 polarization, while M-CSF and CCL2 contribute to M2 polarization. In the present study, we found that TNFα levels were significantly increased in the myometrium, placenta, and decidua at term labor and preterm labor, which contributes to M1 polarization in these tissues. In addition, we found that M2 polarization had a downward trend in most gestational tissues at term and preterm labor. However, the changes in CCL2 and M-CSF levels were not consistent with it. Thus, M2 polarization could be induced by other factors. It should be noted that CCL2 is one of the chemokines that recruit neutrophils and macrophages and some types of T cells to inflamed tissues [60]. Thus, increased CCL2 expression contributes to the influx of these types of immune cells in the gestational tissues. CXCL1 and CXCL2 are the chemokines that recruit not only neutrophils, but macrophages and other immune cells, such as CD8 T cells [61,62]. Thus, the increased expression of CXCL1 and CXCL2 in the myometrium at term labor and RU38486- and LPS-induced preterm labor contributes to the influx of neutrophils, macrophages, and T cells.

As mentioned, LPS acts on TLR4 to activate several signaling pathways such as NF-κB and MAPK, and TLR4 is ubiquitously expressed in various tissues [63]. It has recently been shown that maternal TLR4 signaling is critical for infection-induced preterm birth [13]. In comparison, progesterone maintenance during pregnancy is associated with suppressing NF-κB and AP-1 signaling in gestational tissues [64]. Our previous study has shown that uterine NF-κB signaling is activated at term birth and RU38486- and LPS-induced preterm birth and NF-κB inhibitor can delay term birth as well as RU38486- and LPS-induced preterm labor [9]. In the present study, we have shown a similar pattern of the infiltration of neutrophils and macrophages in the myometrium at term labor and RU38486- and LPS-induced preterm labor, suggesting that these cells are involved in the processes of term and preterm labor. As mentioned, we also found that the IL-1β, IL-6, TNFα, CCL2, CXCL1, and CXCL2 expression was significantly increased in the myometrium at term labor and LPS- and RU38486-induced preterm labor. It is known that these factors are regulated by NF-κB signaling [65,66]. Of note, the population of various leucocytes was not significantly changed in the placenta at term, but it was changed in the placenta at RU38486- and LPS-induced preterm labor, suggesting that fetal reactions are involved in preterm birth as the placenta is one of the fetal tissues. In addition, it seems that intensive inflammatory responses occurred in gestational tissues in LPS-induced preterm birth compared with RU38486-induced preterm birth, as evidenced by the neutrophil and macrophage influx that robustly increased and the expression of most cytokines and chemokines tested, which also robustly increased. Such differences suggest that the mechanisms underlying RU38486-induced preterm labor and LPS-induced preterm birth are different.

As mentioned, NLRP3 inflammasome is involved in physiological and pathophysiological inflammatory responses [67,68]. Several studies have shown that NLRP3 inflammasome activation occurs in gestational tissues at term labor and preterm labor [17,69]. We have recently demonstrated that NLRP3 protein is expressed in human myometrium and fetal membranes, and it is also identified in the smooth muscle cells of the uterus in mice [17]. NLRP3 inflammasome is activated in the uterus at term labor and RU38486-induced preterm labor and LPS-induced preterm birth. The administration of MCC950 prolongs term birth and postpones the preterm labor induced by RU38486 and LPS and suppresses NF-κB activation in the uterus. It is known that NLRP3 inflammasome activation promotes IL-1β and IL-18 production, and subsequently enhances the production of other inflammatory mediators [34]. Thus, uterine NLPR3 inflammasome is also involved in the recruitment of leucocytes. The reduced macrophage infiltration in the uterus caused by MCC950 treatment might be attributed to suppressing NLRP3 inflammasome in the myometrium. In the RU38486 and LPS-induced preterm birth models, MCC950 treatment affected NK cells in the myometrium, decidua, and placenta. Such effects might also be attributed to the changed expression of the chemokines related to the recruitment of NK cells. In fact, details including the subtypes of T cells, B cells, and macrophages as well as the expression of various cytokines in subsets of leukocytes need to be further investigated in our future study.

## 5. Conclusions

The present study showed that both innate and adaptive immune cells were infiltrated in gestational tissues in late gestation in pregnant mice. Macrophages were the largest subset of leukocytes in the myometrium and decidua in late gestation. The influx of macrophages was significantly changed in these tissues at term labor and RU38486- and LPS-induced preterm labor. In late gestation, M2 macrophages were the predominant phenotype in the gestational tissues; however, M1 macrophages were significantly increased in these tissues at term labor and preterm labor. Given that M1 macrophages exhibit proinflammatory function, this suggests that M1 polarization facilitates inflammation linked to the initiation of term labor and preterm labor. Our data immediately provide deep insight into the mechanisms underlying the initiation of term and preterm labor.

## Figures and Tables

**Table 1:** Primers for Q-PCR.

	Forward Primer (5′ -> 3′)	Reverse Primer (5′ -> 3′)
β-actin (Mouse)	CTGTATGCCTCTGGTCGTAC	TGATGTCACGCACGATTTCC
IL-1β (Mouse)	TGCCACCTTTTGACAGTGATG	AAGGTCCACGGGAAAGACAC
IL-6 (Mouse)	CGGCCTTCCCTACTTCACAA	TTCTGCAAGTGCATCATCGT
CXCL1 (Mouse)	CTGGGATTCACCTCAAGAACATC	CAGGGTCAAGGCAAGCCTC
CXCL2 (Mouse)	CCAACCACCAGGCTACAGG	GCGTCACACTCAAGCTCTG
CCL2 (Mouse)	CACCAGCCAACTCTCACTGAA	CATTCCTTCTTGGGGTCAGC
M-CSF (Mouse)	TGATTGGGAATGGACACCTG	AAAGGCAATCTGGCATGAAGT
TNF-α (Mouse)	ACCC TCACACTCAGATCATC	GAGTAGACAAGGTACAA CCC

## Data Availability

Not applicable.
